# Rio+20: Challenges of Development for Health and Sustainability

**DOI:** 10.1289/ehp.1205814

**Published:** 2012-08-31

**Authors:** Maria Cecília de Souza Minayo

**Affiliations:** Editor-in-Chief, *Ciência & Saúde Coletiva*, Rio de Janeiro, Brazil, E-mail: maminayo@terra.com.br

The United Nations Conference on Sustainable Development (Rio+20; United Nations 2011) took place in Rio de Janeiro, Brazil, on 20–22 June 2012. This conference brought together politicians, leaders from social movements, and business representatives from around the world. One goal of the conference was to renew commitment to sustainable development and ensure the promotion of an economically, socially, and environmentally sustainable future for the planet for present and future generations.

*The Future We Want* (United Nations 2012), the zero draft of the outcome document from Rio+20, listed seven priority areas: job creation, food security, water, energy, sustainable cities, oceans, and natural disasters. Unfortunately, as pointed out in a recent editorial from *The Lancet* (2012), human health was hardly mentioned. This is in contrast to Rio-92, where health was viewed as a major theme in Chapter 6 of Agenda 21 (United Nations 1992). *The Lancet* (2012) emphasized the need to ensure that the health agenda of the past decade is completed and that new and emerging concerns that could affect human health, such as noncommunicable diseases and climate change, are considered.

In the past few years, there have been many positive changes in human health and the environment. To demonstrate this point, the June 2012 issue of *Ciência & Saúde Coletiva* (Vol. 17, No. 6; http://www.scielo.br/scielo.php?script=sci_issuetoc&pid=1413-812320120006&lng=en&nrm=iso) was devoted to the changes in human health and the environment in Brazil. Articles in the issue indicated decreases in infant mortality, increases in life expectancy, and decreases in maternal mortality. In addition to these changes, deforestation and burning of forests has declined; the use of technologies that pollute has declined; inspection and surveillance mechanisms relevant to food, water, and air quality have been created; agroecology has expanded; forests have been replanted; and environmental preservations areas have been established. However, Brazil still faces immense environmental liabilities that could affect human health, including the increasing use of agricultural monoculture, continued high-volume use of agricultural pesticides and fertilizers, adverse health impacts from large-scale enterprises (e.g., construction of hydroelectric power plants, sugar and alcohol production, soybean agriculture, raising of livestock), the lack of basic sanitation and solid waste treatment for much of the Brazilian population, increased deforestation and forest burning, the persistence of asbestos and heavy metals, and the unequal distribution of water or lack of clean drinking water.

Many of these health-related issues are not unique to Brazil. In fact, many developed and developing countries face similar environmental challenges that are affecting or could affect human health. Although it is important to promote sustainable development in the future, there is concern about the virtual absence of health-related goals from Rio+20. *Ciência & Saúde Coletiva* endorses the call by *The Lancet* (2012) to move toward integrating health and sustainability goals in any future agenda developed by the United Nations.
